# Fracture Behavior and Toughness Evaluation of Shotcrete: A Closed-Form Approach

**DOI:** 10.3390/ma18112620

**Published:** 2025-06-03

**Authors:** Quan Zhang, Yihuan Xiao, Xiangyu Han, Bin Jia, Kai Zhang

**Affiliations:** 1 School of Civil Engineering, Southwest Jiaotong University, Chengdu 610031, China; quanzhangin@163.com (Q.Z.); 13076079818@163.com (Y.X.); 2 School of Civil Engineering and Architecture, Southwest University of Science and Technology, Mianyang 621010, China; 3 College of Civil and Transportation Engineering, Shenzhen University, Shenzhen 518060, China; zhangkai@szu.edu.cn

**Keywords:** shotcrete, fracture toughness, geometric effect, pore structure

## Abstract

Shotcrete, due to its excellent performance, has become widely adopted as a lining material in tunnel construction. However, research on the fracture behavior of shotcrete, especially in terms of precise fracture toughness determination methods, remains limited. In this study, three distinct batches of shotcrete with varying porosities were fabricated, and their fracture properties were evaluated using three-point bending tests. A closed-form solution was developed to calculate the fracture toughness of shotcrete used in tunnel linings, incorporating factors such as micro-structure, specimen boundaries, and geometry. The results demonstrated that the fracture toughness obtained through this method can be treated as a material constant, independent of specimen geometry. Additionally, the study emphasized the importance of considering the pore structure in the design and fracture analysis of shotcrete materials.

## 1. Introduction

Shotcrete, also called sprayed concrete, is widely applied as the supporting measures in tunnel engineering [1,2,3]. In contrast to regular concrete, shotcrete materials often have various amounts of voids due to the introduction of compressed air during the spraying process [4]. However, these pore structures have a direct impact on the properties of concrete materials and give rise to fracture occurrences under external loads [5]. Consequently, the precise determination of fracture properties is of utmost importance for the effective utilization of shotcrete in tunnel engineering.

Extensive amounts of experimental research have been dedicated to studying the fracture characteristics of shotcrete. Kikkawa et al. [6] applied punching experiments to study the fracture mechanism of shotcrete and established one punching strength formula for early-age shotcrete. Omidi et al. [7] used the Semi-Circular Bend Test and the Notched Brazilian Disc test to investigate the fracture toughness of shotcrete, establishing the relation between fracture toughness and tensile strength. Tayfuner [8] adopted Flattened Brazilian Disc specimens to test the fracture toughness of molded shotcrete, discussing the effects of specimen size and curing time. Lackner and Mang [9] utilized the multi-surface chemoplasticity model formulated in the framework of thermodynamics of chemically reactive porous media to simulate shotcrete and studied the cracking phenomenon in shotcrete tunnel shells. Liu et al. [10] developed a multiscale model to simulate the fracture behavior of freeze-thawed shotcrete; the modeling results were validated by three-point bending test results. Zhang et al. [11] employed bond size theory to establish the discrete element model of steel fiber-reinforced shotcrete, investigating the flexural and fracture performance. It could be found that the fracture test results are consistently influenced by specimen geometries and experimental conditions.

Based on the experimental results, various mechanical models have been proposed to analyze the fracture of concrete materials. Hillerborg [12] used the fictitious crack model to depict the stress distribution around crack tip. Bazant [13] studied the size effect of concrete fracture properties and put forward a size effect law for determining the fracture parameters of concrete. Xu and Reinhardt [14,15,16] divided the fracture process of concrete into two stages (crack initiation and crack propagation) and introduced the double-K fracture criterion to determine the facture parameters. In these models, the influence of specimen geometries on fracture test results is considered, and the accuracy of the determined fracture parameters is always acceptable. Nevertheless, these mechanical models are seldom employed for analyzing the fracture behavior of shotcrete, with the porous micro-structure often being overlooked.

In the present study, fracture tests were conducted on shotcrete materials, and an attempt was made to apply a closed-form solution to analyze the fracture test results of shotcrete specimens with varying conditions. The structure of the current paper is organized as follows: Section 2 outlines the specifics of the manufacturing process and fracture testing of shotcrete and the closed-form solution on the basis of the Boundary Effect Model; Section 3 analyzes the fracture test results; and Section 4 offers a summary of this article.

## 2. Materials and Methods

### 2.1. Materials

**Figure 1:**
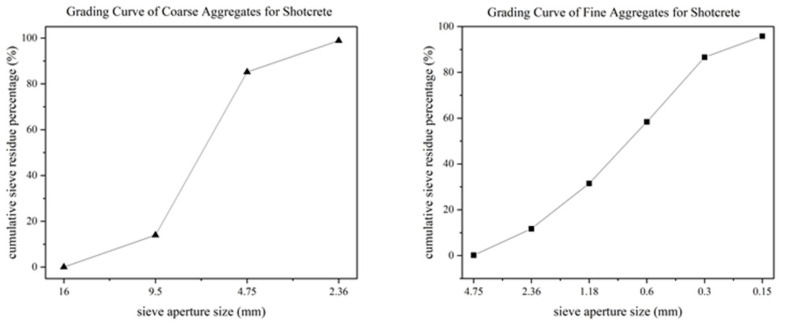
Grading curve of coarse and fine aggregates for shotcrete.

In the present investigation, the composition of shotcrete comprises ordinary Portland cement (Type PO. 42.5, ESheng Cement Ltd., Emeishan City, China), class II fly ash, fine aggregate, coarse aggregate, accelerator, and superplasticizer. Notably, the fine aggregate employed is sourced from natural river sand, exhibiting a fineness modulus of 2.9, while the coarse aggregate is derived from crushed limestone, featuring a maximum aggregate size of 10 mm. Figure 1 illustrates the particle size distribution of coarse and fine aggregates [17]. The precise proportions of the constituent materials, namely cement, fly ash, fine aggregate, coarse aggregate, accelerator, superplasticizer, and water, are meticulously outlined in Table 1. It is imperative to underscore that the introduction of superplasticizer serves the primary purpose of augmenting the flowability of shotcrete, thereby facilitating its conveyance through pipelines. Conversely, the accelerator is strategically incorporated to mitigate the setting time, ensuring that the shotcrete acquires the requisite strength within a condensed temporal frame. In accordance with the provisions of Chinese standards [18,19], the performance parameters of the superplasticizer and accelerator are determined through experiments. Detailed specifications pertaining to the superplasticizer and accelerator can be found in Table 2 and Table 3, respectively.

### 2.2. Method

**Figure 2:**
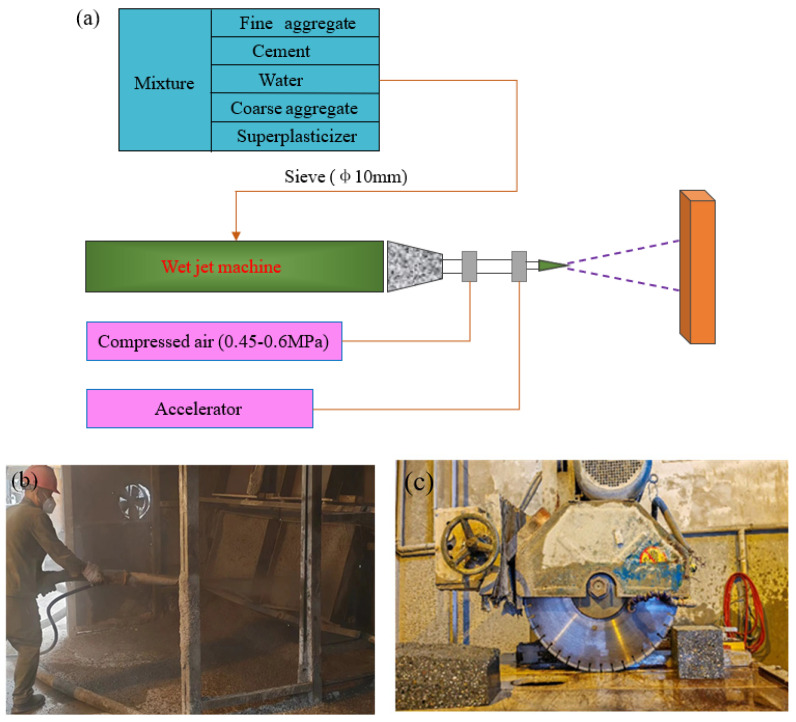
Manufacturing process of shotcrete: (**a**) process diagram; (**b**) spraying scenario; (**c**) specimen cutting.

According to the Chinese National Standard “Technical Specification for Application of Sprayed Concrete” (JGJ/T 372-2016) [20], the production of shotcrete occurs via the wet method and the cured shotcrete slabs are cut into specimens required for the subsequent three-point bending fracture test using a rock cutting machine, as shown in Figure 2. Initially, the cementitious material, aggregates, and superplasticizer are blended using a mixer. Subsequently, the resulting mixture is conveyed to the spraying apparatus, wherein any excessively large aggregates are sieved during this phase. Following this, compressed air (ranging between 0.45 and 0.6 MPa) is generated utilizing an air compressor, and the liquid accelerator is introduced at the nozzle location. Thereafter, the concrete is projected into the molds via the spraying process.

**Figure 3:**
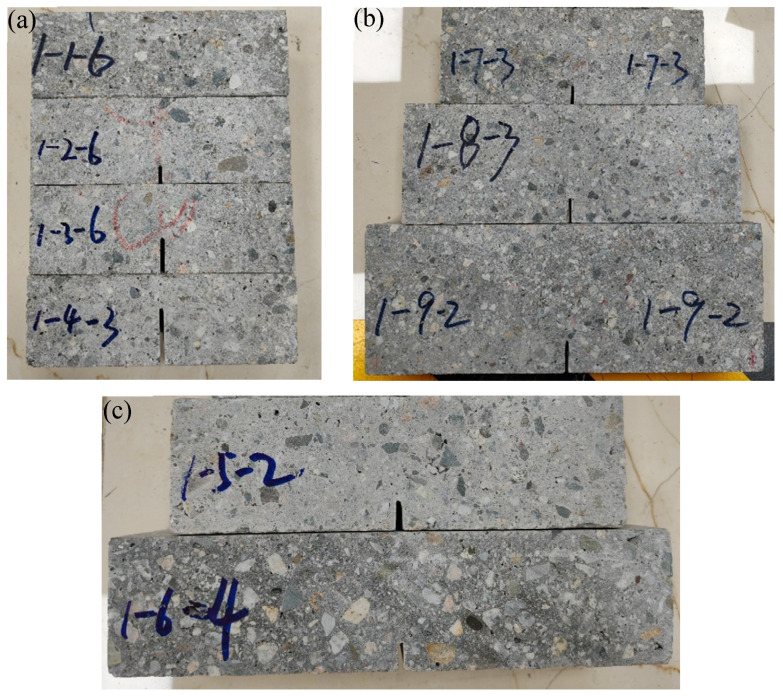
The 3-p-b shotcrete specimens in (**a**) group A; (**b**) group B; and (**c**) group C.

**Figure 4:**
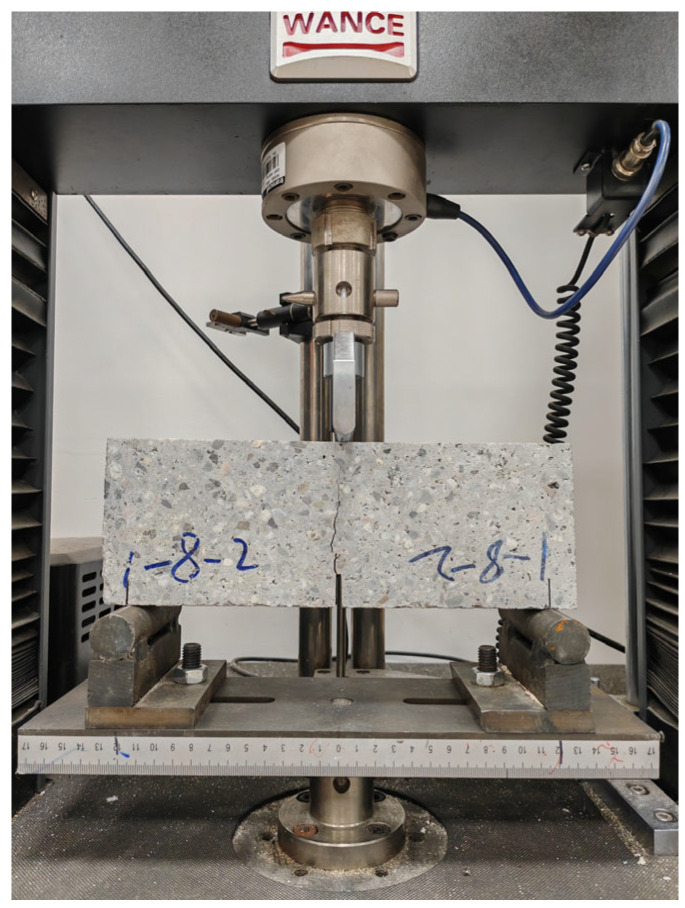
Testing process of shotcrete three-point bending specimen.

After curing, three large slabs are cut into several small samples with different widths, geometries, and initial notch lengths, as depicted in Figure 3. Notably, the thickness of all specimens is standardized at 50 mm. Then, as shown in Figure 4, the three-point bending test (3-p-b) is systematically conducted on these samples at a controlled loading rate of 0.1 mm/min, with the corresponding load–displacement curves meticulously recorded for further analysis [21,22]. As shown in Table 4, the geometric dimensions and average failure loads of specimens in nine groups are summarized.

### 2.3. Analytical Model

In this section, the Boundary Effect Model (BEM) is employed to undertake the fracture analysis of shotcrete specimens. It should be noted that, during the establishment of BEM, considerable attention is dedicated to the effect of specimen size on determining fracture parameters of quasi-brittle materials [23,24,25]. Consequently, the BEM was effectively applied in fracture prediction of concrete, hard rocks, fiber composites, etc. [26,27,28,29,30,31]. The fundamental constituents of BEM are elucidated here.

(1) $$\sigma_{n}=\frac{f_{t}}{\sqrt{1+\frac{a_{e}}{a_{ch}^{*}}}}$$

(2) $$a_{e}={\left[\frac{{\left(1-\alpha \right)}^{2}\cdot Y(\alpha )}{1.12}\right]}^{2}\cdot a_{0}$$

(3) $$\alpha =\frac{a_{0}}{W}$$

(4) $$a_{ch}^{*}=0.25\cdot {\left(\frac{K_{IC}}{f_{t}}\right)}^{2}=3\cdot G_{av}$$

(5) $$\begin{array}{cc} Y_{2.5}(\alpha )=\frac{1-2.5\alpha +4.49\alpha^{2}-3.98\alpha^{3}+1.33\alpha^{4}}{{\left(1-\alpha \right)}^{3/2}} & \left(S/W=2.5\right) \end{array}$$

(6) $$\begin{array}{cc} Y_{4.0}(\alpha )=\frac{1.99-\alpha (1-\alpha )(2.15-3.93\alpha +2.7\alpha^{2})}{\sqrt{\pi }(1+2\alpha ){\left(1-\alpha \right)}^{3/2}} & \left(S/W=4.0\right) \end{array}$$

**Figure 5:**
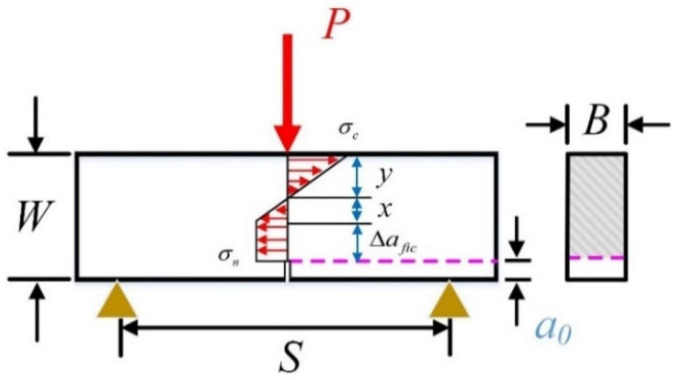
The stress distribution of three-point bending specimens.

Here, the symbol σ_n_ (MPa) represents the nominal stress, while *f*_t_ (MPa) denotes the tensile strength of material. Additionally, *a*_e_ (mm) denotes the equivalent crack length, which can be calculated with initial notch length *a*_0_ (mm), the ratio α of initial notch length *a*_0_ (mm) to the specimen size W (mm), specimen geometry factor *Y*(α). As shown in Figure 5, S is net span of the 3-p-b specimen, then S/W indicates the span-to-height ratio. The characteristic crack $a_{ch}^{*}$ (mm) is a fundamental material parameter that serves to establish the correlation between tensile strength *f*_t_ (MPa) and fracture toughness *K_IC_* (MPa·√m). Moreover, *G*_av_ (mm) is an index for depicting the material micro-structures. Its value is predetermined as half of the maximum aggregate size in the case of ordinary concrete, while for typical rock samples, it is equivalent to the average grain size. In the case of porous materials, the influence of pores necessitates inclusion in the analysis.

The three-point bending test is a widely employed method for assessing the fracture toughness of quasi-brittle materials. As depicted in Figure 5, it is postulated that the stress distribution along the unnotched ligament can be delineated as consisting of linear compressive and linear tensile regions, alongside a constant tensile area, as supported by prior studies [32,33,34]. Subsequently, the relation between maximum failure loads *P_max_* and nominal stress is established, as outlined in Equation (7).

(7) $$\sigma_{n}=\frac{1.5\times \frac{S}{B}\times P_{\max}}{\left(W-a_{0})(W-a_{0}+2\Delta a_{fic}\right)}$$

It is crucial to highlight the significant influence of the fictitious crack in the vicinity of the notch tip on the fracture mechanism of quasi-brittle materials. Ensuring the precise computation of the fictitious crack length ($\Delta a_{fic}$) holds paramount importance in the accurate evaluation of fracture toughness. According to the suggestion in ref. [32], the determination of the fictitious crack length can be effectively achieved using Equation (8), where the effect of notch length, specimen size, pore structures, and micro-structures are all considered.

(8) $$\Delta a_{fic}=\beta \times G_{av}$$

Based on Equations (1), (4), (7), and (8), the relation between fracture toughness and maximum failure loads is effectively modeled and articulated as depicted in Equation (9).

(9) $$K_{IC}=P_{\max}/B_{e}(W,a_{0},G_{av})=P_{\max}\times \frac{3\sqrt{3G_{av}}\times (\frac{S}{B})\times \sqrt{1+\frac{a_{e}}{3G_{av}}}}{\left(W-a_{0})\times (W-a_{0}+2\Delta a_{fic}\right)}$$

## 3. Results

### 3.1. Micro-Structure Index of Porous Shotcrete

**Figure 6:**
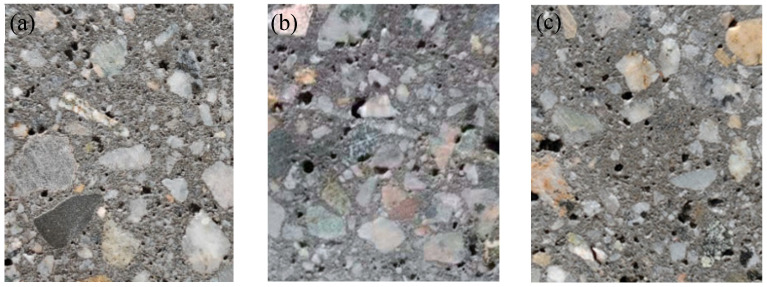
Void distribution of shotcrete specimens in (**a**) group A, (**b**) group B, and (**c**) group C.

Considering the significant impact of micro-structure on the determination of fracture toughness, an initial analysis of the micro-structures of three batches of shotcrete is conducted. As illustrated in Figure 6, 2D images of the shotcrete are collected, revealing the presence of numerous voids within the specimens. This occurrence is primarily attributed to the utilization of compressed air and accelerator during the production process. Consequently, the integration of image processing technology and deep learning algorithms is leveraged to characterize the distribution and volume of these voids, as documented in prior studies [35,36]. To determine the porosity, four representative two-dimensional cross-sectional images were selected for each batch. These images were processed using a Python (3.8)-based image analysis script, in which pores were automatically identified through grayscale thresholding. The porosity index (p) was then calculated accordingly. The resulting porosity values for the three batches of shotcrete were determined to be 0.151, 0.208, and 0.197, respectively. Furthermore, the average aggregate size (G_av_) is conservatively set at half the value of the maximum aggregate size, which is 5 mm.

### 3.2. Determination of Fracture Toughness

Initially, the computation of the fictitious crack length of the shotcrete is paramount and is undertaken in accordance with the principles outlined in Equation (8). In conventional laboratory fracture tests concerning ordinary concrete, the parameter *β* is conventionally designated as 1.5, as established by existing research [37]. However, given the porous nature of the shotcrete materials, it is imperative to account for the influence of pore structures. Consequently, the determination of the fictitious crack length solely takes into consideration the ligament involved in the propagation of tensile cracking phenomena, as elucidated in Equation (10).

(10) $$\Delta a_{fic}=1.5\times G_{av}\times (1-p)$$

Then, the determination of the fracture toughness for each individual shotcrete specimen can be achieved by employing Equation (9). The resultant minimum, maximum, and mean values for each respective group are systematically compiled and presented in a concise manner within Table 5. Notably, pronounced variations are discernible among the fracture toughness values of the specimens, even within the same designated group.

### 3.3. Influence of Initial Notch Length on Fracture Analysis

When subjected to external loads or environmental factors, shotcrete structures can experience fractures of varying magnitudes. Consequently, the analysis of fractured structures becomes imperative. To investigate the influence of initial notch length on fracture analysis, a series of shotcrete specimens in group A, each possessing identical dimensions but differing initial notch lengths, are studied. The fracture toughness values of all specimens in group A are determined using Equation (9) and subsequently subjected to a normal distribution analysis.

**Figure 7:**
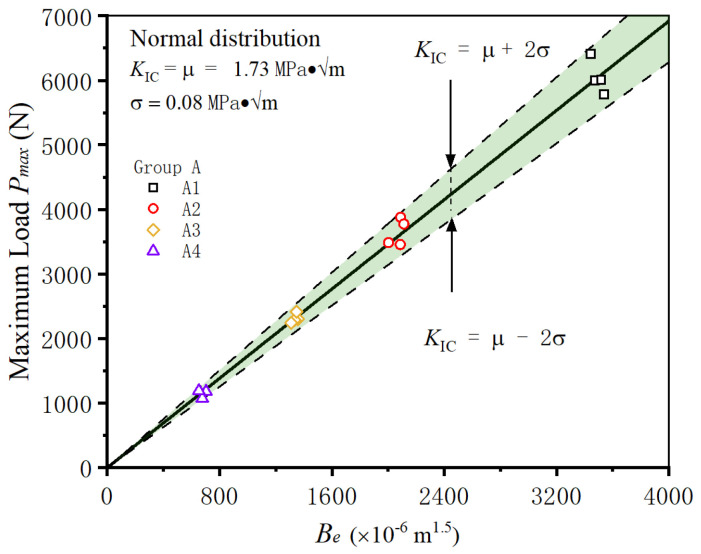
Determining the fracture toughness of shotcrete specimens with different initial notch lengths.

The findings reveal that the fracture toughness of the group A specimens exhibits a mean value of 1.73 MPa·√m, with a standard deviation of 0.08 MPa·√m, representing only 4.6% of the mean value. As shown in Figure 7, plotting all fracture test results and calculation processes of the group A specimens on the same coordinate system reveals a tightly clustered region, indicating that the majority of the data points fell within a narrow range. This suggests that the determined values effectively represent the fracture toughness of the shotcrete in group A, despite the variance in initial notch lengths among the specimens.

### 3.4. Different Specimen Sizes with Single α-Ratio

**Figure 8:**
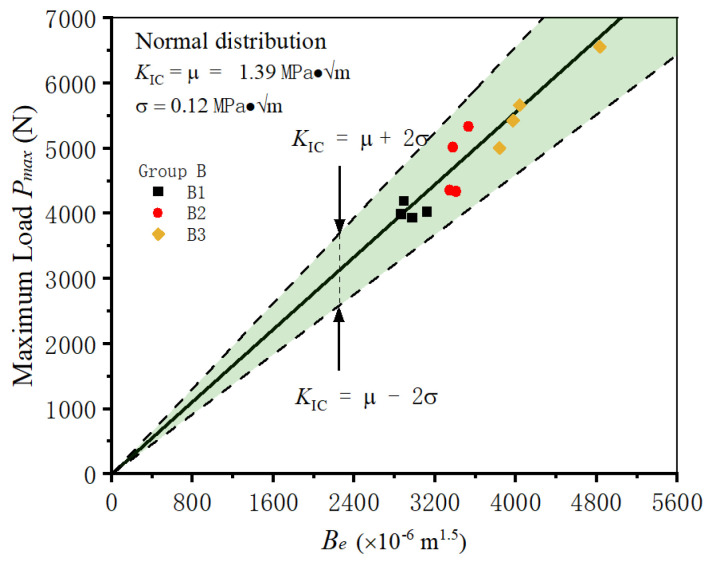
Predicting the fracture of shotcrete specimens with different sizes.

The specimens with different sizes but single α-ratio are always adopted to study the size effect phenomenon of concrete fracture toughness. In group B, the specimens share identical geometrical configurations, featuring an equivalent span-to-width ratio (2.5) and α-ratio (0.2). The widths of three batches of specimens are cut into 80 mm, 100 mm, and 120 mm, respectively. Here, the middle-sized specimens in group B2 are adopted to predict the fracture of samples in the other two groups. Firstly, the distribution of fracture toughness in group B2 is analyzed. Then, the triangle predictive area is constructed with Equation (9) and distribution characteristics, as shown in Figure 8. After that, the fracture test results of groups B1 and B3 are placed in the same coordinates. It could be found that most data in group B1 and B3 fall within the predictive area. This observation strongly indicates the robust predictive capability of the model in anticipating the fracture behavior of specimens within groups B1 and B3, which indicates that the fracture of specimens in groups B1 and B3 are well predicted. With the help of the established model, it is reasonable to ignore the impact of specimen size on the determination of shotcrete fracture toughness, effectively bypassing the size effect phenomenon.

### 3.5. Different Geometries

**Figure 9:**
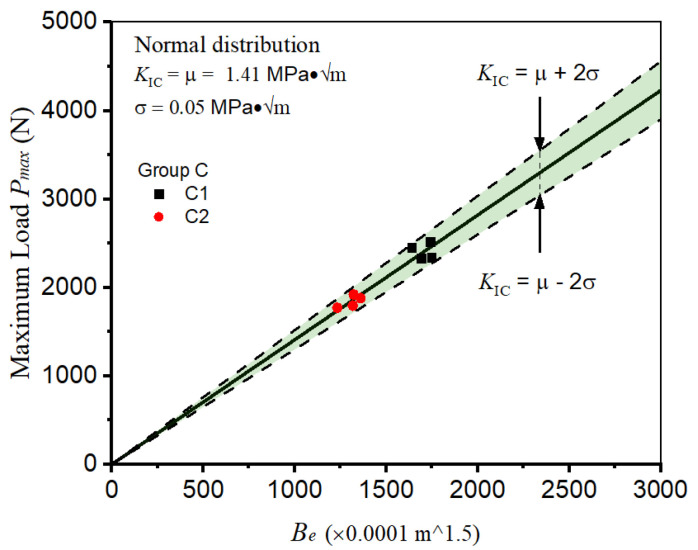
Influence of specimen geometry on calculating shotcrete fracture toughness.

In this section, a comprehensive investigation into the impact of specimen geometry on the computation of fracture toughness is undertaken. Within group C, the specimens possess identical widths and initial notch lengths. However, the span-to-width ratios are deliberately designated as 3.0 and 4.0 in groups C1 and C2, respectively. To accurately evaluate the geometry factor Y(α) for each group, a combination of Equation (5), Equation (6), and an interpolation method is employed. Subsequently, the fracture toughness of each specimen is determined, yielding mean values and standard deviations of 1.41 MPa·√m and 0.054 MPa·√m, respectively. As visually depicted in Figure 9, the distribution of fracture toughness within groups C1 and C2 exhibits striking similarities. Consequently, the calculated fracture toughness can be confidently considered independent of the specific specimen geometries, thereby suggesting the robustness and insensitivity of the fracture toughness calculation methodology to changes in specimen geometries within the defined parameters.

### 3.6. The Influence of Micro-Structures

**Figure 10:**
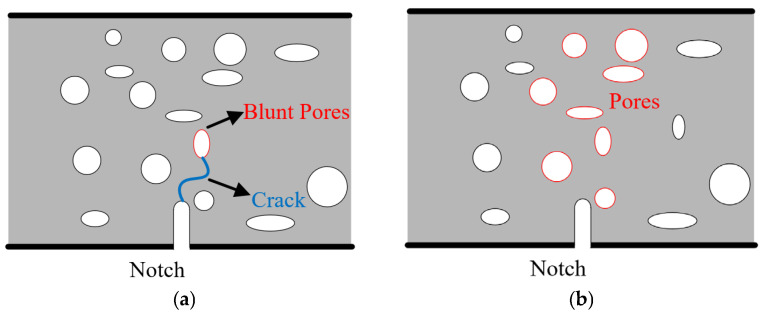
Effect of pores on fracture performance of shotcrete: (**a**) blunting effect; (**b**) decrease in bearing ligament.

The uniformity in mixture design across the three batches of specimens primarily highlights disparities attributed to variations in the air compression pressure during the manufacturing process, consequently leading to discrepancies in pore quantities and sizes. From the aforementioned discussion, it can be observed that the calculated fracture toughness is independent of specimen dimensions, initial crack length, and geometric shape, which means that it can be considered as a material constant. The computed fracture toughness values for groups A, B, and C are 1.73 MPa·√m, 1.37 MPa·√m, and 1.41 MPa·√m, respectively. Evidently, a discernible inverse relationship is discernible, indicating that the fracture toughness of shotcrete diminishes with an increase in porosity. In fact, the influence of pores on the fracture toughness of concrete can be discerned through two distinct facets, as elucidated in Figure 10 [38,39]. On one hand, the presence of pores can effectively blunt the crack tip, thereby postponing crack propagation, reinforcing the material and augmenting its fracture toughness. On the other hand, an escalation in the volume fraction of pores precipitates the weakening of the load-bearing ligament. For the shotcrete materials, the deleterious impact of pores outweighs their reinforcing effects. Consequently, it is imperative to exercise stringent control over the pore content during the shotcrete production process, ensuring that it remains within an optimal and manageable range. Failure to adhere to these guidelines could potentially compromise the fracture toughness of the resulting material.

## 4. Conclusions

Shotcrete has been extensively used in tunnel engineering due to its convenience and mechanical benefits; however, its fracture performance, particularly under the influence of micro-structural features such as porosity, remains underexplored. This study presents a systematic experimental investigation on laboratory-fabricated shotcrete specimens with varying porosities, coupled with an analytical evaluation of fracture toughness using a closed-form solution. The key conclusions are as follows:

(1) A reliable analytical model was developed to estimate the fracture toughness of porous shotcrete materials. The model accounts for critical factors including boundary conditions, specimen geometry, and micro-structural characteristics, and demonstrates good applicability within the linear elastic fracture mechanics framework.
(2) Although experimental data exhibited variability across individual specimens, the calculated fracture toughness within each batch remained nearly constant. This indicates that fracture toughness can be treated as a material property under controlled conditions, largely independent of initial notch length, specimen size, or geometry—thereby verifying the consistency and predictive capability of the proposed model.
(3) The fracture behavior of shotcrete is strongly governed by its internal micro-structure. Increased porosity was found to systematically reduce fracture toughness, highlighting the detrimental effect of voids on fracture resistance. These findings underscore the necessity of incorporating micro-structural considerations—particularly porosity—into both the mix design and mechanical performance evaluation of shotcrete.

The combination of experimental validation and analytical modeling presented in this work enhances the understanding of fracture mechanics in shotcrete. It provides a basis for the further refinement of predictive models and may contribute to the development of performance-optimized shotcrete materials for underground engineering applications.

## Figures and Tables

**Table 1:** Mix proportions of shotcrete (kg/m^3^).

Item	Cement	Fly Ash	Fine Aggregate	Coarse Aggregate	Accelerator	Superpl-asticizer	Water
Content	456	29	875	808	33.95	4.85	182

**Table 2:** Chemical and physical properties of accelerator.

Water-Reducing Rate (%)	Solid Content (%)	Density (g/mL)	Time-Loss of Slump (mm)	Entrained Air Content (%)	Compressive Strength Ratio (%)	
					7d	28d
30	19.6	1.063	25	2.1	153	145

**Table 3:** Chemical and physical properties of superplasticizer.

Major Components	Solid Content (%)	Density (g/mL)	Chloride Ion Content (%)	Alkali Content (%)	Setting Time (s)	
					Initial Setting	Final Setting
Aluminum sulfate	59.5	1.406	0.017	0.05	166	390

**Table 4:** Fracture test results of 3-p-b specimens with various geometries.

Group	Span (mm)	Width (mm)	Initial Notch Length (mm)	Average *P_max_* (N)
A1	125	50	0	6103.2
A2			10	3602.6
A3			20	2300.6
A4			30	1150.2
B1	200	80	16	4128.0
B2	250	100	20	4843.8
B3	300	120	24	5619.0
C1	150	50	10	2419.7
C2	200	50	10	1901.9

**Table 5:** The distribution of calculated fracture toughness.

Group	Fracture Toughness K_IC_ (MPa·√m)		
	Minimum	Maximum	Average
A1	1.63	1.86	1.73
A2	1.66	1.86	1.76
A3	1.69	1.79	1.71
A4	1.58	1.82	1.70
B1	1.29	1.45	1.36
B2	1.27	1.51	1.39
B3	1.30	1.40	1.36
C1	1.33	1.49	1.41
C2	1.36	1.45	1.41

## Data Availability

The original contributions presented in this study are included in the article. Further inquiries can be directed to the corresponding authors.
